# Expanding the clinical and mutational spectrum of *B4GALT7*-spondylodysplastic Ehlers-Danlos syndrome

**DOI:** 10.1186/s13023-017-0704-3

**Published:** 2017-09-07

**Authors:** Marco Ritelli, Chiara Dordoni, Valeria Cinquina, Marina Venturini, Piergiacomo Calzavara-Pinton, Marina Colombi

**Affiliations:** 1 Division of Biology and Genetics, Department of Molecular and Translational Medicine, School of Medicine, University of Brescia, Viale Europa 11, 25123 Brescia, Italy; 2 Division of Dermatology, Department of Clinical and Experimental Sciences, Spedali Civili University Hospital, Brescia, Italy

**Keywords:** Spondylodysplastic Ehlers-Danlos syndrome, *B4GALT7*, Larsen of Reunion Island syndrome, Linkerophaties

## Abstract

**Background:**

Spondylodysplastic EDS (spEDS) is a rare connective tissue disorder that groups the phenotypes caused by biallelic *B4GALT7*, *B3GALT6,* and *SLC39A13* mutations. In the 2017 EDS nosology, minimal criteria (general and gene-specific) for a clinical suspicion of spEDS have been proposed, but molecular analysis is required to reach a definite diagnosis. The majority of spEDS patients presented with short stature, skin hyperextensibility, facial dysmorphisms, peculiar radiological findings, muscle hypotonia and joint laxity and/or its complications. To date only 7 patients with β4GALT7-deficiency (spEDS-*B4GALT7*) have been described and their clinical data suggested that, in addition to short stature and muscle hypotonia, radioulnar synostosis, hypermetropia, and delayed cognitive development might be a hallmark of this specific type of spEDS. Additional 22 patients affected with an overlapping phenotype, i.e., Larsen of Reunion Island syndrome, all carrying a homozygous *B4GALT7* mutation, are also recognized.

**Results:**

Herein, we report on a 30-year-old Moroccan woman who fitted the minimal criteria to suspect spEDS, but lacked radioulnar synostosis and intellectual disability and presented with neurosensorial hearing loss and limb edema of lymphatic origin. Sanger sequencing of *B4GALT7* was performed since the evaluation of the spEDS gene-specific minor criteria suggested this specific subtype. Mutational screening revealed the homozygous c.829G>T, p.Glu277* pathogenetic variant leading to aberrant splicing.

**Conclusions:**

Our findings expand both the clinical and mutational spectrum of this ultrarare connective tissue disorder. The comparison of the patient’s features with those of the other spEDS and Larsen of Reunion Island syndrome patients reported up to now offers future perspectives for spEDS nosology and clinical research in this field.

## Introduction

Spondylodysplastic Ehlers-Danlos syndrome (spEDS) is a rare autosomal recessive connective tissue disorder with an unknown frequency and prevalence. In the 2017 revised EDS classification, spEDS groups the phenotypes caused by biallelic *B4GALT7*, *B3GALT6,* and *SLC39A13* mutations within the same clinical entity in consideration of the reliable clinical overlap [1]. Two major criteria, i.e., short stature and muscle hypotonia, plus characteristic radiographic abnormalities and at least three other minor criteria are minimal criteria suggestive for spEDS [1, 2]. Confirmatory molecular testing is mandatory to reach a final diagnosis. *B4GALT7* and *B3GALT6* encode galactosyltransferase I (β4GALT7) and II (β3GALT6), respectively, that are Golgi-resident enzymes involved in synthesizing the glycosaminoglycan (GAG) linker region of proteoglycans [3]; *SLC39A13* encodes the trans-membrane Zrt/irt like protein 13 (ZIP13) that regulates the influx of zinc into the cytosol [4].

At present, 7 patients from 6 families with molecularly confirmed spEDS due to β4Galt7-deficiency (spEDS-*B4GALT7*) have been reported and a total of 7 missense and 2 frameshift mutations are recognized [3, 5–8]. All or the majority of reported patients with spEDS-*B4GALT7* showed short stature, muscle hypotonia, radioulnar synostosis, and mild to severe intellectual disability (ID), consequently, these items were assumed to be the hallmark of the disorder [1, 2, 7]. Other frequent features comprised facial dysmorphism, hyperextensible skin, joint hypermobility (JHM), single transverse palmar crease, severe hypermetropia, limb bowing, and osteopenia, [2]. Further 22 patients, all with the same homozygous *B4GALT7* p.(Arg270Cys) missense mutation and strict clinical overlap with spEDS-*B4GALT7*, have been characterized in the ethnic group called white creoles living on Reunion Island (Larsen of Reunion Island syndrome, LRS) [9].

Herein we describe an additional patient with a novel homozygous *B4GALT7* causative variant and compare her clinical features with those of the other spEDS and LRS patients reported so far, thus expanding the phenotype of the disorder and its allelic repertoire.

## Patient report

The proposita, a 30-year-old Moroccan woman, was born from healthy, apparently non-consanguineous parents and had a healthy younger sister. Perinatal distress was absent. At birth, measurements were within normal range; neonatal hypotonia and slight delayed motor development were noted. In infancy, progressive height deficit not related to GH deficiency was present, and a clinical diagnosis of unspecified EDS was given for skin hyperextensibility and generalized JHM. At age 28, dual-energy X-ray absorptiometry disclosed low bone mineral density for sex and age (z-score < 2 SD); audiometric test revealed mild neurosensorial hearing loss. Genetic analyses were not performed.

**Fig. 1:**
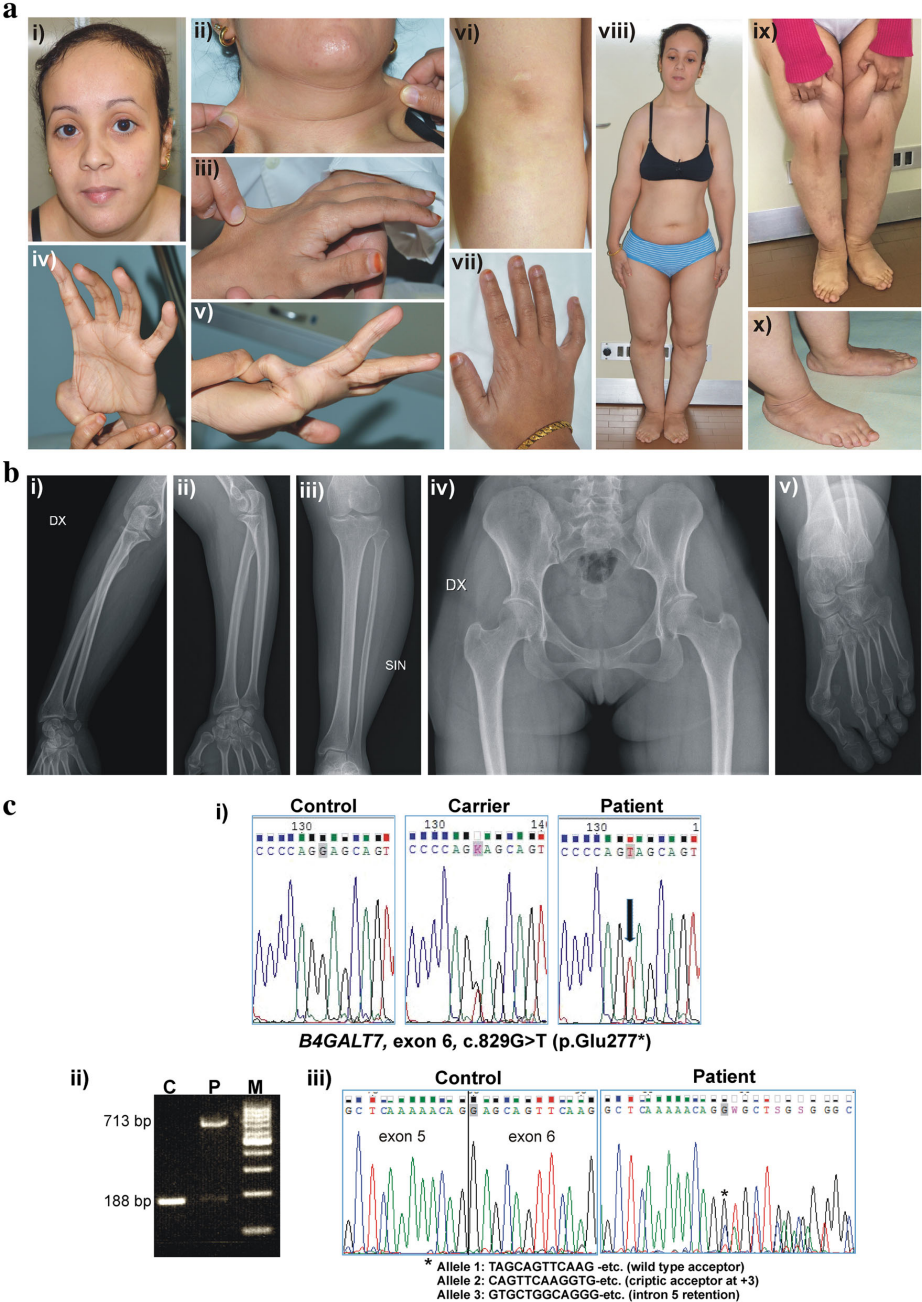
Clinical and molecular findings of the patient. **a** Facial features, i.e., light blue sclerae, wide forehead, flat face, sparse scalp hair, narrow mouth (i), skin hyperextensibility on the neck and dorsum of the hand (ii, iii), single palmar crease and laxity of the thumb (iv), laxity of the fifth finger (v), small atrophic scar on knee (vi), low-set thumb and clinodactyly of the fifth finger (vii), disproportionate short stature, hypotonia and edema of the lower limbs (viii, ix), pes planus and hallux valgus (x). **b** Absence of radioulnar synostosis (i-iii), osteopenia and absence of metaphyseal flaring (iv), metatarsophalangeal subluxation and severe hallux valgus (v). **c** Sequence chromatograms showing the position of the homozygous *B4GALT7* c.829G>T variant (arrow) (seq. Ref.: NM_007255.2, NP_009186.1) (i). RT-PCR with a primer pair encompassing exons 4–6 performed on total RNA from patient’s blood demonstrated that the c.829G>T transversion, which affects the first nucleotide of exon 6, impacts splicing (ii). In particular, apart from the usage of the wild type acceptor (allele 1), an alternative acceptor 3 bp downstream (allele 2), and the presence of a splice product with retention of intron 5 (allele 3) were disclosed (iii). These different alleles should lead to a truncated protein (p.Glu277*), in-frame deletion (p.Glu277del), and insertion of 10 amino acids followed by a stop codon (p.Glu276_Glu277ins11*), respectively

On examination, disproportionate short stature (145 cm, genetic target 160 cm, standard deviation from ethnic average stature – 2, arm span/height ratio 1.09, n.v. <1.05), soft, doughy, hyperextensible skin, small atrophic scars on knees and a single transverse palmar crease were observed (Fig. 1A). gJHM according to the Beighton score (7/9), skeletal abnormalities (scoliosis, severe hallux valgus, pes planus, low-set thumb, clinodactyly of the 5th fingers), generalized muscle hypotonia, facial dysmorphism (light blue sclerae, wide forehead, flat face, sparse scalp hair, and narrow mouth), and edema of the lower extremities were also noticed (Fig.1A). Complication of gJHM were recurrent sprains, temporomandibular joint dislocation and chronic pain at cervical spine, right shoulder, and ankles. Doppler ultrasound of lower limbs established edema of lymphatic origin; lymphography was not performed according to the patient’s choice. The most frequent causes of secondary lymphedema, i.e., cancer, infection, and surgery, were not present. Ophthalmologic evaluation was unremarkable for refractive errors including hypermetropia. Forearms, left wrist, pelvis, lower limbs, feet and spine X-ray disclosed dextroscoliosis, L5 hemisacralitation, L4-L5 disc space narrowing, metatarsophalangeal subluxation, pes planus, and hallux valgus (Fig. 1B). Heart ultrasound detected normal cardiac/valve morphology and function. Cognitive development and mentation were normal.

This phenotype was suggestive for spEDS, since the patient fulfilled 2 major and 4 minor criteria according to the 2017 EDS nosology (Table 1), and the evaluation of the gene-specific minor criteria suggested *B4GALT7* as causative gene (Table 2).

**Table 1:** Major and minor criteria of spEDS according to the 2017 EDS nosology [1, 2] and comparison to LRS [9]

	Present patient	spEDS-*B4GALT7* (%)^a^	spEDS-*B3GALT6 (%)*	spEDS-*SLC39A13 (%)*	Total spEDS (%)	LRS (%)
MAJOR CRITERIA						
Short stature	+	8/8 (100)	26/36 (72.2)	7/8 (87.5)	41/52 (78.8)	19/19 (100)
Muscle hypotonia	+	8/8 (100)	16/36 (44.4)	6/8 (75.0)	30/52 (57.6)	na
Bowing of limbs	–	5/8 (62.5)	3/36 (8.3)	na	8/44 (18.1)	–
MINOR CRITERIA						
Hyperextensible, soft, doughy, and thin skin	+	7/8 (87.5)	19/36 (52.7)	6/8 (75.0)	32/52 (61.5)	21/22 (95.4)
Pes planus/equinovarus/valgus	+	6/8 (75.0)	21/36 (58.3)	6/8 (75.0)	33/52 (63.4)	na
Delayed motor development	+	7/8 (87.5)	7/36 (19.4)	–	14/52 (26.9)	na
Osteopenia	+	5/8 (62.5)	12/36 (33.3)	3/8 (37.5)	20/52 (38.4)	–
Delayed cognitive development	–	5/8 (62.5)	11/36 (30.5)	–	16/52 (30.7)	12/22 (54.5)

*na* not available

^a^present patient included

**Table 2:**
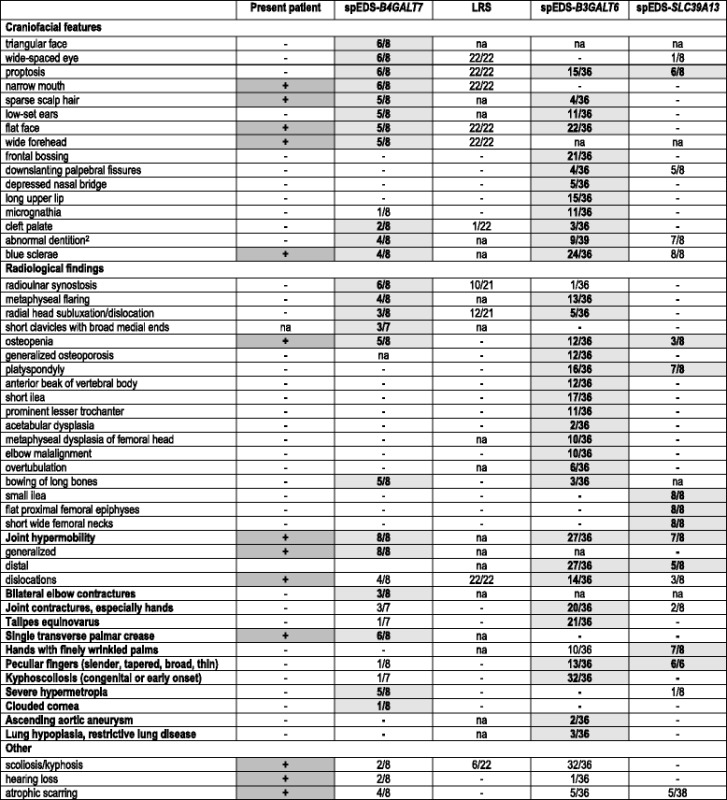
Gene-specific minor criteria and other features of spEDS according to the 2017 EDS nosology [1, 2] and comparison to LRS [9]

^1^present patient included; ^2^tooth discoloration, dysplastic teeth; na: not available. The gene-specific minor criteria for all types of spEDs are indicated in bold and in light grey, in dark gray the signs observed in the present patient

Therefore, after written informed consent was obtained, *B4GALT7* mutational screening was performed by Sanger sequencing of all coding exons/intron boundaries. This analysis disclosed in the patient the homozygous c.829G>T (p.Glu277*) variant; both parents and her sister were heterozygotes (Fig. 1C). All these carriers did not show any sign of the disease including short stature, muscle hypotonia, craniofacial dysmorphism, as well as hearing loss and lymphedema.

The identified variant represents a very rare allele seen only twice in 122,600 samples from the genome Aggregation Database (gnomAD) with an allele frequency of 8.157e^−6^. Though patient's parents firmly denied consanguinity, the presence of some not-so-distant relatedness should not be excluded; homozygosity mapping was not performed, since they did not consent further investigations. Alternatively, the allele might be more common in Morocco, but no population-specific data are available. The transversion affects the first nucleotide of the last exon and the splice site prediction tools of the Alamut Visual version 2.9.0 software suggested that the variant alters the canonical splice acceptor site. To verify the possible splice effect of the variant, RT-PCR was carried out by standard procedures on RNA extracted from patient’s whole blood. That demonstrated multiple splice outcomes. In particular, agarose gel electrophoresis of patient’s cDNA amplified with primers encompassing exons 4–6 showed, in addition to low amounts of wild type fragment (188 bp), the presence of an aberrant band (713 bp). Sanger sequencing of this RT-PCR showed in addition to mRNA resulting from the use of the canonical site, the presence of 2 further splice products: i) an allele derived from the activation of a cryptic splice acceptor site 3 bp downstream generating in-frame deletion of the amino acid residue at position 277, and ii) mRNA with entire intron 5 retention causing the insertion of 10 amino acids followed by a stop codon (Fig. 1C). The mutation should therefore result in different amounts of truncated (p.Glu277* and p.Glu276_Glu277ins11*) and internally deleted polypeptides (p.Glu277del) leading to reduced galactosyltransferase activity. The resulting functional implication on GAG synthesis was not studied, since fibroblasts were not available.

## Discussion

spEDS-*B4GALT7* is an extremely rare and consequently poorly characterized entity. In the past, nosologic confusion characterized this disorder, since patients with *B4GALT7* mutations were alternatively labelled as EDS progeroid type [5, 6], EDS with short stature and limb anomalies [7], linkeropathy [3], and LRS [9]. The term “progeroid” was originally used due to the phenotypic resemblance including mild aged appearance, which was observed among patients with β4Galt7-deficiency of the first reports [5, 6] and those categorized as EDS progeroid variant by Hernandez et al. [10–12]. Description of further patients did not confirm the association of β4Galt7-deficiency with early aging [7–9]. The umbrella term linkeropathies refers to the overlapping phenotypes caused by mutations in genes encoding for the enzymes involved in the synthesis of the GAG linker region that include, apart from *B4GALT7* and *B3GALT6*, also *B3GAT3* and *XYLT1/2* encoding glucuronyltransferase I and xylosyltransferase I/II, respectively [13].

In the 2017 EDS nosology, the entity “EDS progeroid type” is no longer listed and *B4GALT7* and *B3GALT6*-related linkeropathies were grouped as spEDS together with patients harboring *SLC39A13* mutations [1]. Patients with *B3GAT3* and *XYLT1/2* mutations were not classified as spEDS even if numerous common features are recognized, i.e., short stature, muscle hypotonia, cutaneous signs, gJHM, characteristic radiographic findings (foot abnormalities, peculiar fingers, swedish key appearance of femora, metaphyseal widening, radioulnar synostosis), and facial dysmorphism (prominent forehead, blue sclerae, proptosis, small mouth, and depressed nasal bridge) [13–15].

Minimal criteria to suspect spEDS have been proposed based on the clinical findings of reported patients [2]. Concerning spEDS-*B4GALT7* clinical data of 7 molecularly characterized EDS patients were considered, whereas the 22 LRS patients were not included. Consistent with the proposed criteria, our patient further reinforces the concept that short stature and muscle hypotonia really seem the prominent features of spEDS-*B4GALT7*, since they have been observed in all patients described so far. Considering all the spEDS types, these two features were present in about 80 and 58%, respectively. Severe short stature has been disclosed also in LRS patients, while data concerning muscle hypotonia were not available (Table 1). Bowing of limbs, the third major criterion of spEDS, was reported only in about 18% of spEDS patients, whereas it was found more frequently in spEDS-*B4GALT7*, suggesting that this sign might be more specific of this subtype of spEDS (Table 1, [2]). Other frequent features described in most spEDS-*B4GALT7* patients include gJHM, skin hyperextensibility, radioulnar synostosis, severe hypermetropia, and facial dysmorphisms (Tables 1 and 2). Skin hyperextensibility, radioulnar synostosis and similar facial dysmorphisms have been recognized also in patients with LRS (Tables 1 and 2). Beighton score in LRS patients was not reported, however, all patients presented multiple dislocations highly suggestive for JHM (Table 1, [9]). Platyspondyly, a hallmark of “spondylo”-dysplasia, has never been recognized in spEDS-*B4GALT7* and in LRS, whereas it is frequently reported in spEDS-*B3GALT6* and spEDS-*SLC39A13* (Table 2). Given the strict clinical overlap among LRS and spEDS-*B4GALT7,* in our opinion*,* these conditions should not be considered as distinct entities. The presence of the same p.(Arg270Cys) mutation in both disorders [6–9] further supports this hypothesis. Nevertheless, some phenotypic variations between LRS and spEDS-*B4GALT7* exist, such as presence of bifid thumb, brachymesophalangy, and glaucoma and absence of hypermetropia and osteopenia in LRS [9]. These differences may stem from the high level of homozygosity among the LRS population and/or modification by interactions with other variants in linkage disequilibrium to *B4GALT7* [8, 9].

The recently defined gene-specific minor criteria are undoubtedly helpful to address molecular analysis, since some features such as facial dysmorphism and radiological findings have been described only among patients with a specific subtype (Table 2). Indeed, the clinical presentation of our patient was suggestive for *B4GALT7*-deficiency, since she presented narrow mouth, wide forehead, and single transverse palmar crease that were described only in spEDS-*B4GALT7*. In addition, she showed some common gene-specific minor criteria, i.e., blue sclerae, sparse scalp hair, flat face, osteopenia, and joint hypermobility, but none of those that are unique of the other types (Table 2). Even if only few spEDS-*B4GALT7* patients are reported, a wide spectrum of phenotypic severity associated with β4Galt7-deficiency seems to exist, suggesting that patients with milder phenotypes may lack some features that have been associated with the syndrome. For instance, our patient did not show some assumed hallmark of the disorder, i.e., radioulnar synostosis and ID, and other frequent reported features including proptosis, limb bowing, and hypermetropia (Table 1). It is possible that the present patient’s milder features are due to a less deleterious effect of her pathogenetic variant that might retain more galactosyltransferase activity as compared to the previously reported variants.

Our patient presented mild neurosensorial hearing loss, reported up to now only in another spEDS-*B4GALT7* patient and in an individual with *B3GALT6*-deficiency [Table 2], and lower limb edema of lymphatic origin, never described before in spEDS. Presence of hearing loss in 2/8 spEDS-*B4GALT7* patients suggests that hearing screening should be included in patients’ evaluation, at least until hearing impairment will not be better defined by further reports. Concerning lower limb lymphedema, though it has been reported in a 12-month-old boy born to a consanguineous couple with a homozygous *B3GAT3* mutation [14], data are too scarce to speculate if this sign might be associated with spEDS and overlapping disorders such as linkeropathies. Given that we were not able to rule out some level of consanguinity of patient’s parents, other alleles could play a role in the pathogenesis of both hearing loss and lymphedema.

In conclusion, further reports are needed to better characterize the *B4GALT7*-related phenotypes, particularly, to define the incidence of the assumed specific features, delineate genotype-phenotype correlations, outline if LRS (and possibly also patients with *B3GAT3* and *XYLT1/2* mutations) should be considered as part of spEDS phenotypic spectrum, collect natural history data for prognostication, and to reach a critical number of patients for therapeutic studies.

## Abbreviations

ID: Intellectual disability
JHM: Joint hypermobility
LRS: Larsen of Reunion Island syndrome
spEDS: spondylodysplastic Ehlers-Danlos syndrome

## References

[1] Malfait F, Francomano C, Byers P (2017). The 2017 international classification of the Ehlers-Danlos syndromes. Am J Med Genet C.

[2] Brady AF, Demirdas S, Fournel-Gigleux S (2017). The Ehlers-Danlos syndromes, rare types. Am J Med Genet C..

[3] Arunrut T, Sabbadini M, Jain M, Machol K, Scaglia F, Slavotinek A (2016). Corneal clouding, cataract, and colobomas with a novel missense mutation in B4GALT7—a review of eye anomalies in the linkeropathy syndromes. Am J Med Genet A.

[4] Bin BH, Fukada T, Hosaka T (2011). Biochemical characterization of human ZIP13 protein: a homodimerized zinc transporter involved in the spondylocheiro dysplastic Ehlers–Danlos syndrome. J Biol Chem.

[5] Kresse H, Rosthoj S, Quentin E (1987). 1987. Glycosaminoglycan-free small proteoglycan core protein is secreted by fibroblasts from a patient with a syndrome resembling progeroid. Am J Hum Genet.

[6] Faiyaz-Ul-Haque M, Zaidi SH, Al-Ali M (2004). A novel missense mutation in the galactosyltransferase-I (B4GALT7) gene in a family exhibiting facioskeletal anomalies and Ehlers-Danlos syndrome resembling the progeroid type. Am J Med Genet A.

[7] Guo MH, Stoler J, Lui J (2013). Redefining the progeroid form of Ehlers-Danlos syndrome: report of the fourth patient with B4GALT7 deficiency and review of the literature. Am J Med Genet A.

[8] Salter CG, Davies JH, Moon RJ (2016). Further defining the phenotypic spectrum of B4GALT7 mutations. Am J Med Genet A.

[9] Cartault F, Munier P, Jacquemont ML (2015). Expanding the clinical spectrum of B4GALT7 deficiency: homozygous p.R270C Mutation with founder effect causes Larsen of Reunion Island syndrome. Eur J Hum Genet.

[10] Hernández A, Aguirre-Negrete MG, Ramírez-Soltero S (1979). A distinct variant of the Ehlers-Danlos syndrome. Clin Genet.

[11] Hernández A, Aguirre-Negrete MG, Liparoli JC, Cantú JM (1981). Third case of a distinct variant of the Ehlers-Danlos syndrome (EDS). Clin Genet.

[12] Hernández A, Aguirre-Negrete MG, González-Flores S (1986). Ehlers-Danlos features with progeroid facies and mild mental retardation. Further delineation of the syndrome. Clin Genet.

[13] Taylan F, Mäkitie O (2016). Abnormal proteoglycan synthesis due to gene defects causes skeletal diseases with overlapping phenotypes. Horm Metab Res.

[14] Jones KL, Schwarze U, Adam MP, Byers PH, Mefford HC (2015). A homozygous B3GAT3 mutation causes a severe syndrome with multiple fractures, expanding the phenotype of linkeropathy syndromes. Am J Med Genet A.

[15] Job F, Mizumoto S, Smith L (2016). Functional validation of novel compound heterozygous variants in B3GAT3 resulting in severe osteopenia and fractures: expanding the disease phenotype. BMC Med Genet.

